# Role of Tad pili during the transition from planktonic to biofilm state in *Bradyrhizobium diazoefficiens* USDA 110

**DOI:** 10.1128/jb.00008-25

**Published:** 2026-01-12

**Authors:** J. Iglesias, D. Colla, J. S. Serrangeli, M. J. Lozano, O. Falduti, D. Brignoli, I. Medici, M. J. Althabegoiti, A. R. Lodeiro, P. L. Abdian, N. Paczia, A. Becker, A. Soler-Bistué, J. Perez-Gimenez, E. J. Mongiardini

**Affiliations:** 1 Facultad de Ciencias Exactas, Instituto de Biotecnología y Biología Molecular (IBBM), Universidad Nacional de La Plata y CCT—La Plata, CONICET 172355 , La Plata, Argentina; 2 Curso de Genética, Departamento de Ciencias Biológicas, Facultad de Ciencias Agrarias y Forestales, Universidad Nacional de La Plata 117147 , La Plata, Argentina; 3 Instituto de Investigaciones Biotecnológicas, IIBio, Universidad Nacional de San Martín- Consejo Nacional de Investigaciones Científicas y Técnicas (CONICET) 63042 , San Martín, Provincia de Buenos Aires, Argentina; 4 Instituto de Microbiología y Zoología Agrícola (IMyZA) G.V. al IABIMO, INTA—CONICET, Hurlingham, Buenos Aires, Argentina; 5 Max Planck Institute for Terrestrial Microbiology 28310, Marburg, Germany; 6 Center for Synthetic Microbiology (SYNMIKRO), Marburg, Germany; 7 Department of Biology, Philipps-Universität Marburg 9377, Marburg, Hesse, Germany; National Institutes of Health, Bethesda, Maryland, USA

**Keywords:** *Bradyrhizobium*, biofilms, pili, c-di-GMP, symbiosis, soybean, surface adhesion

## Abstract

Free-living soil bacteria can exist in two main states: planktonic, as motile single cells, or sessile, within biofilms. In biofilms, bacterial cells are embedded in an extracellular matrix that provides protection from environmental stresses and enhances long-term survival. The transition from planktonic to biofilm states sometimes involves surface sensing and attachment, processes commonly mediated by flagella and pili. In this study, we investigated the role of Type IVc Tad pili in surface sensing, adhesion, and biofilm formation in *Bradyrhizobium diazoefficiens*, a nitrogen-fixing symbiont of soybean. Bioinformatic analyses revealed that Tad pili are widely distributed and highly conserved within the *Bradyrhizobium* genus. While pili deletion in other model organisms typically reduces biofilm formation, we found that deletion of the most conserved genomic cluster encoding Tad pili in *B. diazoefficiens* led to increased adhesion to abiotic surfaces and impaired motility—indicative of a physiological shift toward a biofilm-associated state. These findings suggest that Tad pili may play a sensory or regulatory role, potentially influencing cell-cell or cell-matrix interactions. Furthermore, we identified a link between Tad pili and intracellular c-di-GMP levels. Together, these results highlight the critical role of Tad pili in the physiology of *B. diazoefficiens* and offer new insights into bacterial surface adaptation, with potential applications in agriculture and biotechnology. Understanding these mechanisms is essential for improving biofilm management strategies and developing new approaches to enhance bacterial survival in soil and inoculant formulations, ultimately optimizing legume symbiosis.

**IMPORTANCE:**

Biofilm formation is essential for bacterial survival in soil environments. In this study, we investigated the role of Tad pili in the biofilm-forming capacity of *Bradyrhizobium diazoefficiens* and their connection to the second messenger c-di-GMP, a key regulator of the transition between planktonic and sessile states. Bacteria used in agricultural inoculants are typically in the planktonic state, yet survival and persistence are optimized in the sessile state. Our findings may contribute to the development of strategies that promote the transition to the biofilm lifestyle in inoculant formulations, thereby enhancing bacterial viability in storage and soil and improving symbiotic performance with host plants.

## INTRODUCTION

Free-living soil bacteria exist in two main states: planktonic and sessile. Planktonic bacteria are characterized as individual cells that can swim freely. In contrast, sessile bacteria often aggregate into biofilms—structured communities in which cells adhere to each other and produce an extracellular matrix, developing multicellular traits that result in a highly organized and resilient structure (1–3). Biofilms can form on solid surfaces, at liquid–air interfaces, or even as floating aggregates in liquid environments. Within biofilms, bacterial cells are embedded in a polymeric matrix composed of polysaccharides, proteins, and/or nucleic acids, which provides protection from environmental stress and enhances cell survival (4).

During the transition from planktonic to biofilm lifestyles, particularly when the biofilm forms on a surface, surface sensing is typically mediated by bacterial appendages such as flagella and pili, which play critical roles in initiating biofilm formation (5, 6). Flagella are primarily responsible for motility, enabling bacteria to reach favorable environments, whereas pili are filamentous structures involved in a range of functions, including adhesion and surface interaction (7, 8).

Pili, also referred to as fimbriae, are fibers composed of pilin subunits that extend outside the cell. Particularly, the Type IV pili (T4P) have the ability to polymerize and depolymerize the pilin subunits, allowing extension and retraction of the filament. This dynamic behavior enables bacteria to explore surfaces, attach, and establish cohesive communities (9, 10). Multiple types of pili have been identified across bacterial species, each associated with distinct cellular functions (11, 12). The functional versatility of pili underscores their importance in bacterial survival and interactions with both biotic and abiotic environments.

Pili are classified into several families based on their structural and functional characteristics. Among these, T4P are the most abundant and diverse, being found across both Eubacteria and Archaea (11). T4P are involved in various processes, including twitching motility, DNA uptake during natural transformation, and virulence in pathogenic bacteria (9, 13, 14). Within this group, the Type IVc subclass (T4cP), also known as Tad pili, is particularly noteworthy. Tad pili are widely distributed and are implicated in surface sensing, adhesion, and biofilm development (15–17).

Tad pili are encoded within genomic islands, and their pilin subunits typically exhibit low molecular mass (15). They were first described in *Aggregatibacter actinomycetemcomitans* as responsible for tight adherence to surfaces, which gave them the name “Tad” (tight adherence) pili (18). This adhesive capability supports the formation of robust biofilms, which are essential for colonization and persistence in the oral cavity, contributing to periodontal disease (19). In *Caulobacter crescentus*, Tad pili were initially identified as a system that is regulated with the cell cycle progression (20), and more recently, have been identified as one of the primary systems involved in surface sensing (21). Together with the flagellum, these pili initiate a signaling cascade upon surface contact, which drives the developmental transition to the biofilm mode of life (22–25). In other bacteria, such as *Agrobacterium tumefaciens*, Tad pili are essential for adhesion to abiotic surfaces and biofilm formation, processes critical for plant host colonization (26). Similarly, in *Sinorhizobium meliloti* and *Bradyrhizobium diazoefficiens*, Tad pili are involved in early interactions with plant roots during symbiosis establishment, influencing bacterial competitiveness in forming nitrogen-fixing nodules (27–29).

Another key regulator of the transition between planktonic and sessile lifestyles is the second messenger cyclic di-GMP (c-di-GMP), a signaling molecule that integrates a variety of environmental and cellular cues (30). Upon surface contact, c-di-GMP levels rise, triggering the genetic and physiological programs required for biofilm development. The synthesis of c-di-GMP is catalyzed by diguanylate cyclases, which are often functionally linked to flagellar and pilus systems. Mechanical disruption of these appendages by surface contact acts as a trigger for c-di-GMP production (21, 22).

In the genus *Bradyrhizobium*, which comprises nitrogen-fixing bacteria that form symbiotic nodules in legume plant roots, the presence and role of pili have been previously noted (31). Unlike other legume-associated rhizobia, *Bradyrhizobium* species are typically slow-growing, possess large genomes, and can nodulate a wide range of hosts, often showing remarkable adaptation to acidic soils and environmental stresses. *B. diazoefficiens* has been characterized as possessing adhesive fimbriae; however, neither its physiological role has been explored yet, nor the specific structural gene encoding the 20 kDa pilin subunit has been identified (31). In a previous study, we identified a *tadG* homolog in *B. diazoefficiens*, suggesting a possible connection to Tad pili (29). This protein is part of a small gene cluster with high homology to known accessory proteins or pseudopilins. Nevertheless, its involvement in Tad pilus structure and function has not been confirmed.

In this study, we present a phylogenetic analysis of the Tad pilus apparatus in the *Bradyrhizobium* genus, with a focus on its role in regulating the transition from planktonic to sessile states. By characterizing the genetic and structural components of the Tad pili in *B. diazoefficiens*, we aim to elucidate their function in surface sensing, adhesion, and biofilm formation. Additionally, we explore the link between Tad pili and c-di-GMP signaling. Understanding the regulation of bacterial adhesion mechanisms is crucial not only for advancing our knowledge of bacterial physiology but also for developing applications in agriculture and biotechnology, where manipulating biofilm formation could offer significant benefits.

## RESULTS

### Tad pilus is widely present and highly conserved in the *Bradyrhizobium* genus

The T4cP (21) is widely distributed among *Bradyrhizobium* species. A recent study reported that *B. diazoefficiens* USDA 110 harbors four distinct Tad pilus gene clusters (28), which we renamed clusters 1 through 4 based on their chromosomal order according to the pair base coordinates. In this study, we carried out a bioinformatic and molecular characterization of these clusters in *Bradyrhizobium*.

**Fig 1:**
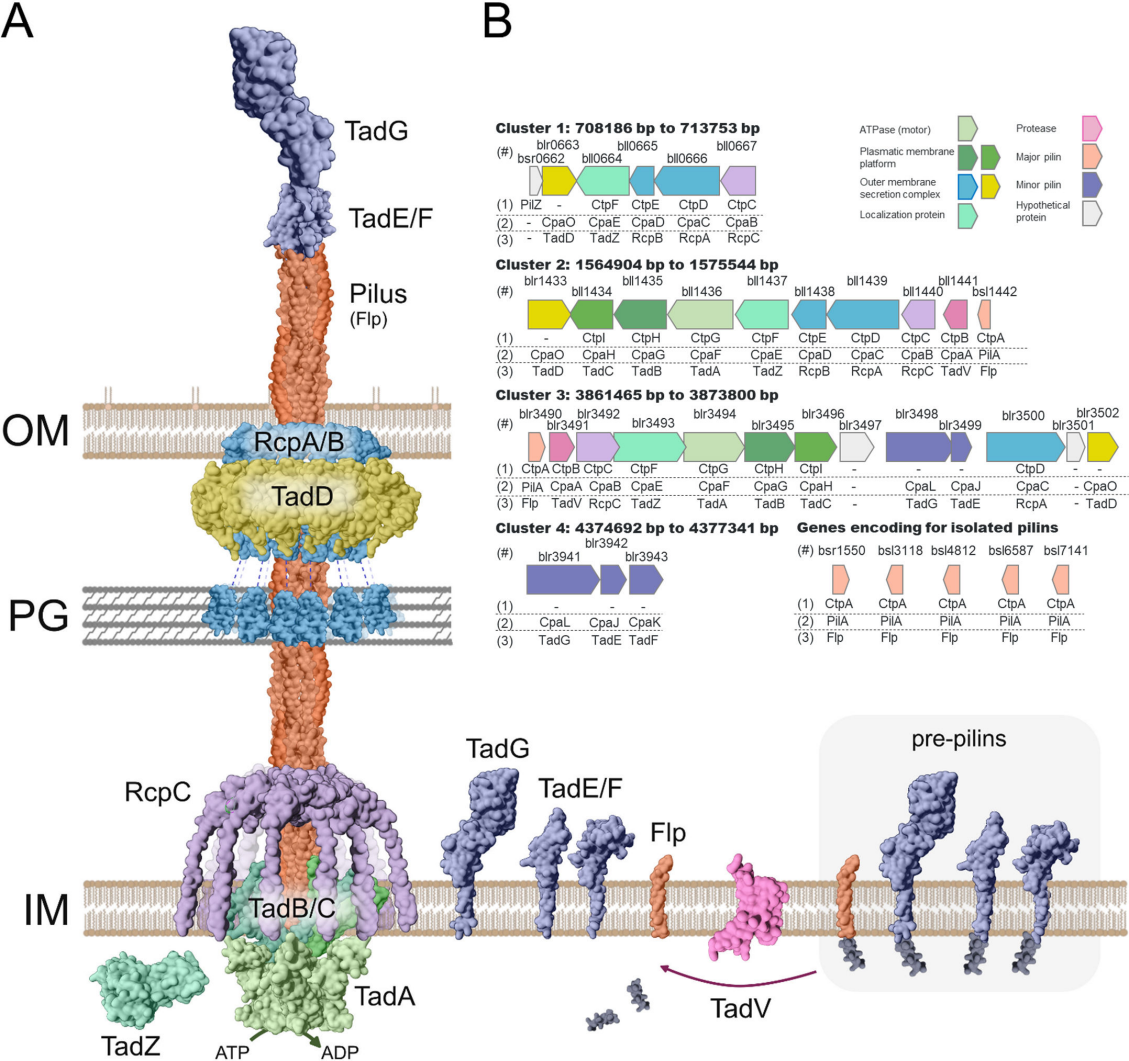
Tad pili clusters encoded in *Bradyrhizobium diazoefficiens* USDA 110. (**A**) Scheme of a Tad pilus. (**B**) Scheme of the gene clusters related to Tad pili systems in *B. diazoefficiens* USDA 110. The genes assigned to each cluster are indicated below the gene, according to the results shown in Table S1 (http://sedici.unlp.edu.ar/handle/10915/186424), and using the same nomenclature as in three different bacterial models: (1) *Agrobacterium tumefaciens* C58, (2) *Caulobacter crescentus* N1000, and (3) *Actinobacillus actinomycetemcomitans* D7S-1. (#) Each gene is identified by the locus tag (above each gene box).

*B. diazoefficiens* USDA 110 possesses two major clusters that encode the full set of genes required for assembly of the Tad export apparatus (Fig. 1A), and two minor clusters that encode only partial pilus-related functions. For consistency, genes within each cluster were annotated using nomenclature derived from *A. tumefaciens* C58 (26), *C. crescentus* NA1000 (20), and *A. actinomycetemcomitans* D7S-1 (19). Gene functions were predicted based on sequence similarity using reciprocal BLASTp analysis (Fig. 1B; see Table S1 at http://sedici.unlp.edu.ar/handle/10915/186424). When multiple paralogs were present, the corresponding cluster number was appended as a suffix.

To compare the four clusters of *B. diazoefficiens* USDA 110 with those characterized in other microorganisms, we used the Clinker algorithm (see Fig. S1 at http://sedici.unlp.edu.ar/handle/10915/186424). Within *B. diazoefficiens*, we observed a high degree of similarity between cluster 2 and the partially encoded cluster 1, suggesting that cluster 1 may have originated from a duplication of cluster 2. Additionally, cluster 2 exhibited strong conservation in gene sequence and organization with homologous clusters from *A. tumefaciens*, *S. meliloti*, and *C. crescentus*. In contrast, clusters 3 and 4 showed lower conservation relative to the other clusters 1 and 2.

To further investigate the evolutionary origin of cluster 3, we constructed a phylogenetic tree based on concatenated alignments of TadA, TadB, and TadC proteins, following the approach used by Ellison et al. (32) and including homologs from clusters 2 and 3 (see Fig. S2A at http://sedici.unlp.edu.ar/handle/10915/186424). The resulting tree supported the Clinker-based findings: clusters 2 and 3 belong to the same clade but form distinct branches (see Fig. S2B at http://sedici.unlp.edu.ar/handle/10915/186424). Cluster 2 groups with *C. crescentus*, *A. tumefaciens*, and *S. meliloti*, while cluster 3 groups with *Vibrio*, *Aggregatibacter*, and *Azospirillum* species (see Fig. S2A and B at http://sedici.unlp.edu.ar/handle/10915/186424). Additionally, a separate phylogenetic analysis based on TadA protein sequences from a broader set of species (from Ellison et al. [32]), including TadA sequences from clusters 2 and 3 of *B. diazoefficiens*, yielded similar groupings (see Fig. S2C at http://sedici.unlp.edu.ar/handle/10915/186424). These findings suggest that clusters 2 and 3 did not originate via gene duplication but rather arose through distinct evolutionary events.

We also examined all *Bradyrhizobium* genome sequences available in the NCBI database (1,505 genomes, accessed on 30/09/2024; see Table S2 at http://sedici.unlp.edu.ar/handle/10915/186424). Our analysis revealed that nearly all genomes contain multiple Tad pilus clusters, encoding either a complete or partial pilus exporter apparatus (see Fig. S3 at http://sedici.unlp.edu.ar/handle/10915/186424). Notably, 95% of the genomes encode clusters 1, 2, and 4 (fully or partially). In the remaining 5%, where Tad pili could not be detected, genomes were available only as contig-level or scaffold-level assemblies, which may explain the lack of detection. To visualize the distribution of Tad clusters, we mapped their presence onto a phylogenomic tree constructed using FastANI and a representative set of diverse *Bradyrhizobium* species (see Table S3 at http://sedici.unlp.edu.ar/handle/10915/186424). The analysis showed that most species harbor complete or partial versions of clusters 1, 2, and 4, while cluster 3 appeared to be sporadically distributed across the genus (see Fig. S4; Table S3 at http://sedici.unlp.edu.ar/handle/10915/186424). These results support the hypothesis that cluster 1 may have originated from a duplication of cluster 2 in a common ancestor of *Bradyrhizobium*, which is consistent with the observed conservation and synteny (see Fig. S1 to S3 at http://sedici.unlp.edu.ar/handle/10915/186424 and phylogenetic tree).

Although cluster 4 was absent in its canonical form in 51.5% of the analyzed genomes (see Table S2 at http://sedici.unlp.edu.ar/handle/10915/186424), protein-level analysis revealed that TadG is present in 95% of them (see Fig. S3 at http://sedici.unlp.edu.ar/handle/10915/186424), and a similar prevalence was observed for TadE and TadF. This suggests that while cluster 4 may not be conserved in terms of genomic arrangement, its genes are widely distributed and possibly functionally retained. These genes are required for pilus biosynthesis and likely play a regulatory role in the formation of a fully functional pilus (33). Altogether, our findings support the notion that the Tad pilus system is an important and conserved feature in *Bradyrhizobium* biology. However, its functional role in this genus remains to be fully elucidated.

### Tad pilus affects biofilm formation in *B. diazoefficiens* USDA 110

**Fig 2:**
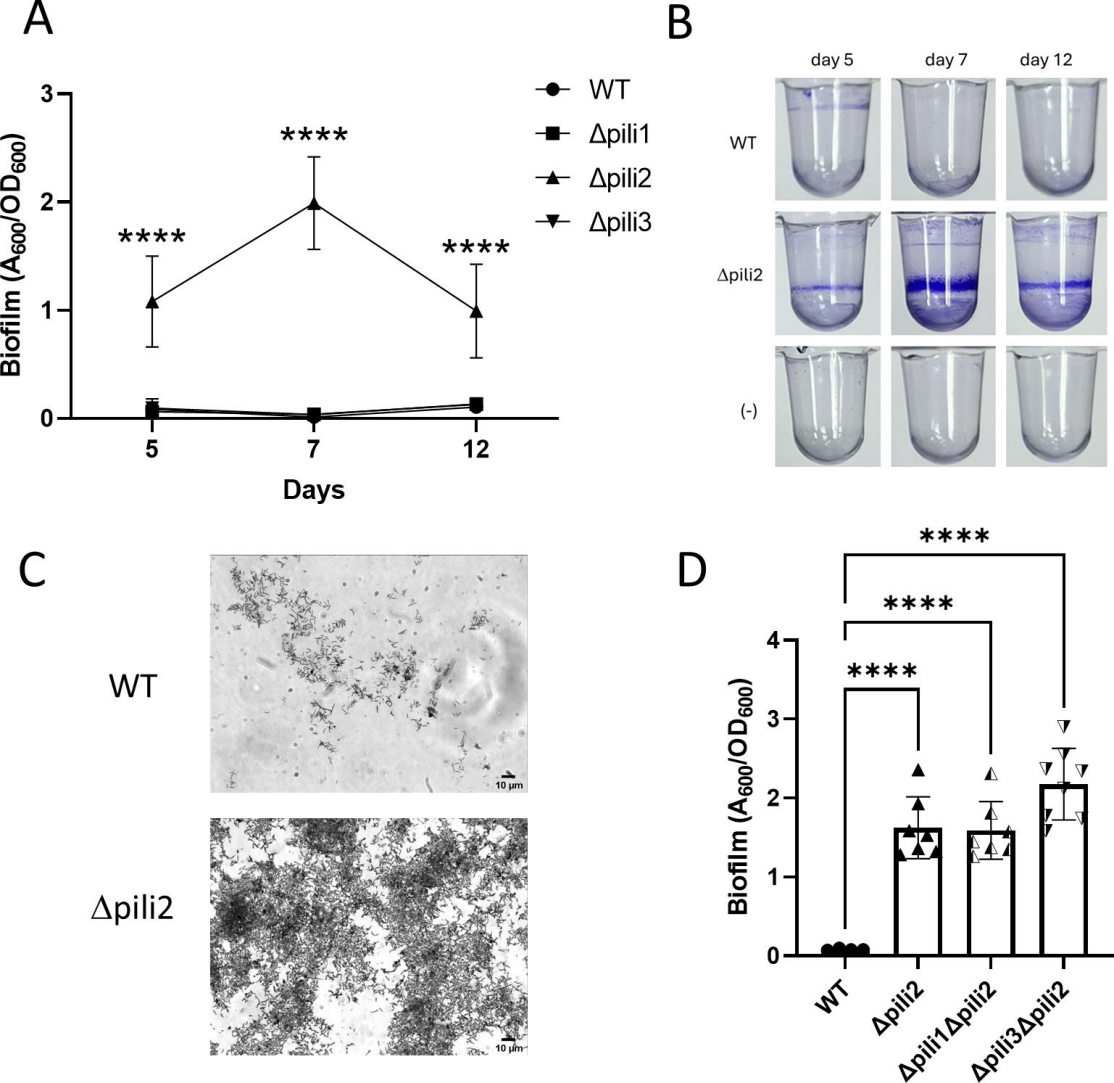
Biofilm formation by WT and Δpili mutants. (**A**) Evaluation of biofilm formation at different incubation times. Data represent the results from two independent experiments, each with four biological replicates. (**B**) Representative images of wells stained with CV for the WT and the Δpili2 mutant before quantification. (**C**) WT and Δpili2 mutant observed by light microscopy after CV staining. (**D**) Biofilm formation was evaluated for the double mutants Δpili1Δpili2 and Δpili3Δpili2 compared to the WT and the Δpili2 mutant after 7 days incubation time. Statistical significance was determined by analysis of variance and Tukey’s *post hoc* test comparison for differences between strains indicated. ****, *P* < 0.0001; only statistically significant differences have been indicated.

Wide conservation of these clusters suggested the physiological and ecological importance of pili in *Bradyrhizobium*. To unravel the role of pili in *B. diazoefficiens* USDA 110, we generated deletion mutants of pili clusters 1, 2, and 3 (TP1, TP2, and TP3) (see Fig. S5 at http://sedici.unlp.edu.ar/handle/10915/186424). Cluster 4 (TP4) was not mutagenized because we studied it in earlier work (29). The mutants were tested for biofilm formation capacity, measured as adhesion in polyvinyl chloride (PVC) 96-well microtiter plates (Fig. 2A and B). The Δpili2 mutant showed a 12-fold to 20-fold increase in biofilm formation compared to the wild type (WT). This was unexpected, as these types of pili are usually associated with adhesion to surfaces. In most of the bacterial models studied to date, lack of Tad pili leads to impairment of adhesion and biofilm formation (26, 34–40). Besides, the other two mutants, namely Δpili1 and Δpili3, exhibited a behavior similar to the WT (Fig. 2A and B). These findings were confirmed by testing several clones obtained independently from different merodiploids, and in all cases, identical results were obtained (data not shown). A similar phenotype of increased biofilm formation was confirmed by direct observation of 7-day biofilms formed on a glass slide under light microscopy after crystal violet (CV) staining (Fig. 2C).

To explain this observation, we hypothesized that the augmented biofilm formation capacity in the Δpili2 mutant may be the result of deregulation or upregulation of TP3 when TP2 loses its function (41). To test this, we evaluated the biofilm formed by double mutants where TP1 and TP2 (Δpili1Δpili2) or TP2 and TP3 (Δpili3Δpili2) were genetically removed. Interestingly, and similarly to the single Δpili2 mutant, both double mutants increased their biofilm-forming capacity (Fig. 2D). Since cluster 1 does not encode all the components required to assemble a functional secretion apparatus and lacks an associated pilin gene, it is unlikely to be capable of producing a complete and functional pilus on its own. Therefore, the Δpili3Δpili2 double mutant should not be able to form a functional apparatus. Since it displays the same phenotype as the Δpili2 single mutant, this supports the idea that the Δpili2 deletion is responsible for the observed phenotype.

During the analysis of biofilm formation, we observed a peculiar behavior of the Δpili2 mutant that could be linked to its phenotype of increased adhesion to PVC. Unlike the WT strain, the Δpili2 mutant does not sediment at the bottom of the well. Figure S6 (http://sedici.unlp.edu.ar/handle/10915/186424) shows WT, Δpili1, and Δpili3 strains forming compact bacterial sediments at the bottom of the well after 7 days of incubation (42). However, this effect was not observed in the Δpili2 mutant. This phenomenon could be directly related to the function of Tad pili, which facilitates interactions between cells and the formation of aggregates, as observed in *A. actinomycetemcomitans* (43). The fact that the Δpili2 mutant sediments at a lower rate supports the hypothesis that this strain has lost its functional pili apparatus.

**Fig 3:**
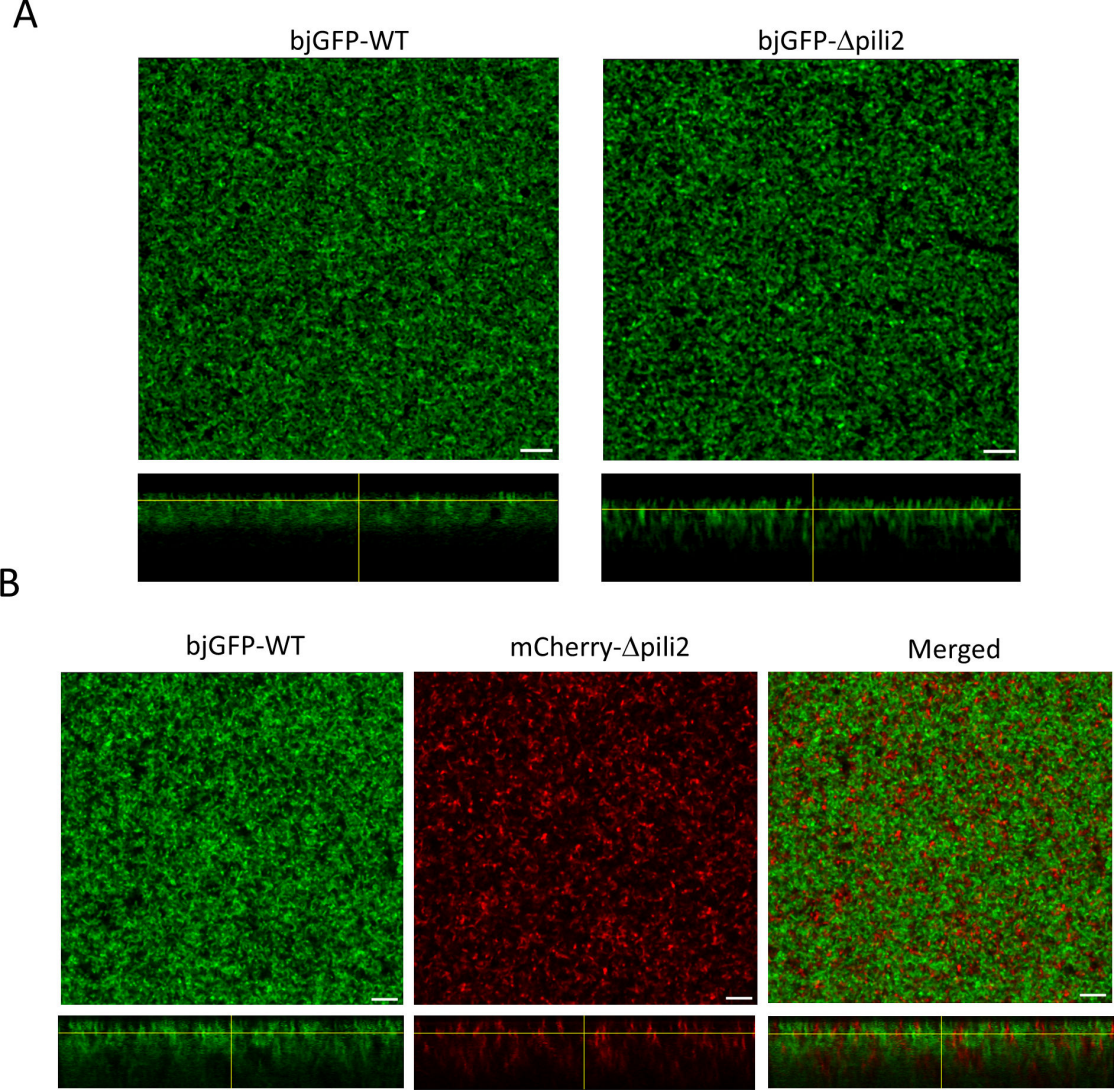
Biofilm structure analysis. CSLM images of horizontal (XY) and vertical (XZ) projections (large and bottom panels, respectively) of 7-day biofilms formed by WT and the Δpili2 mutant. (**A**) Individual biofilms formed by bjGFP-derived WT and Δpili2 strains. (**B**) Mixed biofilm formed by bjGFP-WT (green) and mCherry-Δpili2 mutant (red). No interactions between the WT and mutant were observed inside the cell clusters (merged image). The glass surface is in the upper side of the XZ projections; scale bars represent 10 µm. Each assay was repeated with at least two biological replicates.

In addition, the biofilms formed by fluorescent derivatives of the WT and Δpili2 strains were evaluated by confocal scanning laser microscopy (CLSM) at day 7. Overall, the biofilm architecture formed by these strains on chambered cover glass appeared slightly different (Fig. 3A). However, XZ projections showed that the WT developed a dense biofilm structure, unlike the Δpili2 mutant, which formed loosely packed macro-aggregates, resulting in a biofilm crossed by multiple pores and channels. When bjGFP-WT (green) and mCherry-Δpili2 (red) were allowed to form a mixed biofilm at 1:1 ratio (Fig. 3B), the architecture of each structure was modified in comparison to the individual biofilms. Interestingly, both strains appeared to segregate within the mixed biofilm in a pattern similar to that commonly observed when different species are co-cultured, forming distinct clusters composed exclusively of either WT or Δpili2 mutant cells, as is more clearly observed in the cross-sectional view. This observation highlights the critical role of Tad pili in the recognition and establishment of tight cell-cell interactions, supporting the notion that the loss of cell-cell interaction observed in the Δpili2 mutant is directly linked to the absence of these structures. It is important to note that the rates of biofilm formation by bacteria expressing any of the fluorescent labels (bjGFP or mCherry) were similar (data not shown), and no differences in biofilm formation, as measured by multiwell assay, were detected between biofilms formed by fluorescently labeled and unlabeled bacteria.

### Lack of Tad pili2 impairs bacterial motility

Biofilm formation and motility are often inversely regulated in bacteria, forming part of mutually exclusive developmental programs. Given the contrasting biofilm phenotypes observed among the Tad pilus mutants, we next assessed whether motility was also affected in these strains.

**Fig 4:**
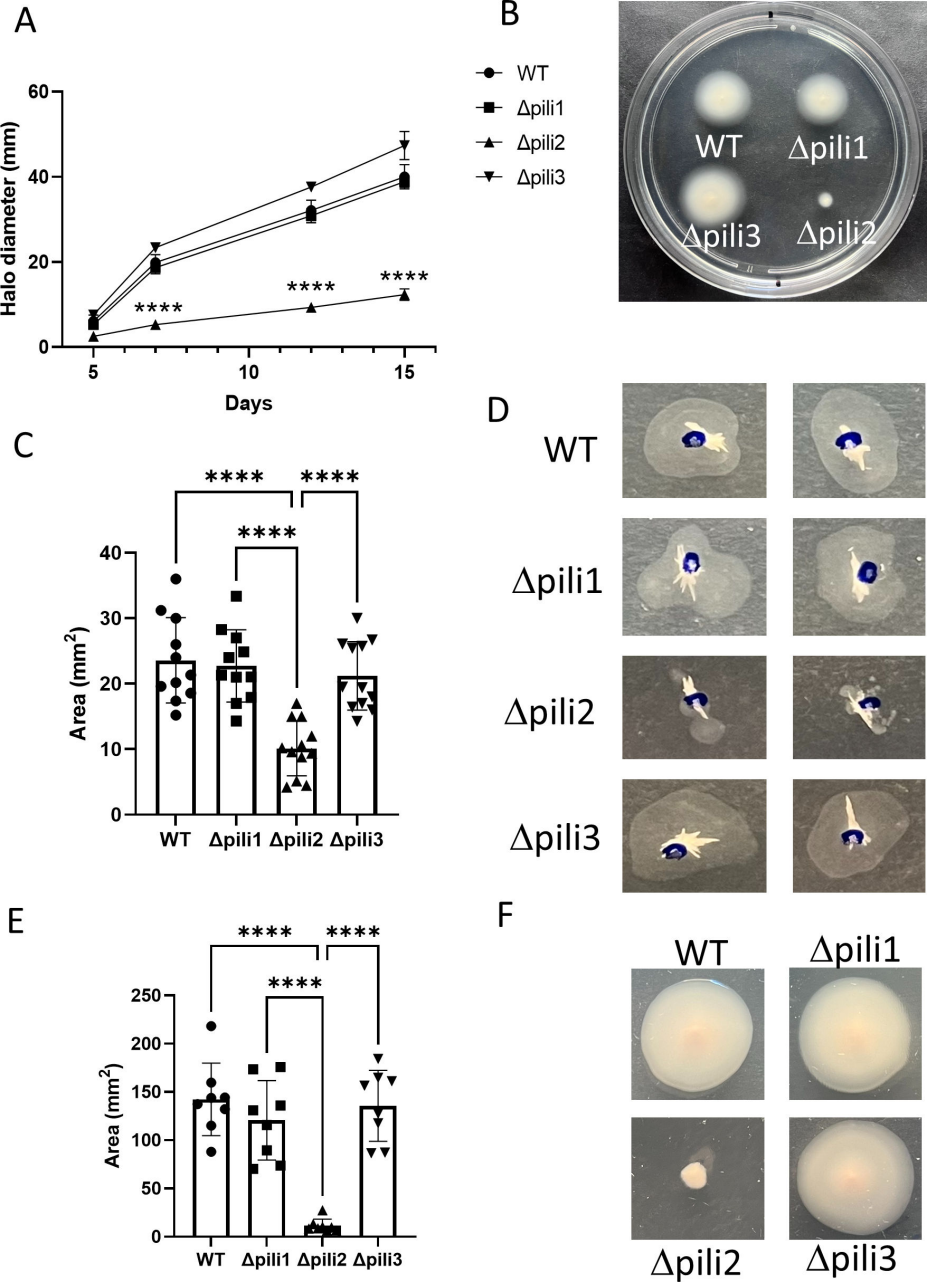
Motility characterization of the pili mutants. (**A**) Swimming in semisolid AG medium (0.3% agar) over time. (**B**) Swimming plate after 7 days incubation with the WT and pili mutants. (**C**) Area covered by twitching motility in the interface between the growth medium and the plate (AG medium, 0.8% agar). (**D**) Two representative images for each strain from the twitching experiment. (**E**) Area covered by surface-associated motility (SAM) over the top of the growth medium (AG medium, 0.8% agar). (**F**) One representative image for each strain of the experiment of SAM. Data represent results from at least two independent experiments, each with a minimum of four biological replicates. Statistical significance was determined by analysis of variance and Tukey’s *post hoc* test comparisons for differences between strains. ****, *P* < 0.0001; only statistically significant differences were indicated.

First, we evaluated swimming motility on semisolid medium (0.3% agar). The Δpili2 mutant exhibited a pronounced reduction in swimming ability compared to the WT, Δpili1, and Δpili3 strains (Fig. 4A and B). To investigate whether this motility defect was due to altered flagellar structure or expression, we purified flagellins from liquid cultures and analyzed them by SDS-PAGE (see Fig. S7 at http://sedici.unlp.edu.ar/handle/10915/186424). *B. diazoefficiens* USDA 110 expresses two independent flagellar systems—subpolar and lateral—both of which contribute to swimming motility in soft agar (44). The SDS-PAGE analysis revealed the presence of both high-molecular-weight FliC1234 (subpolar flagellins) and low-molecular-weight LafA12 (lateral flagellins). Although the Δpili2 mutant exhibited a reduced amount of lateral flagellin compared to WT, this alone does not account for the swimming deficiency, since ΔLafA mutants are still capable of swimming via the subpolar flagellum (44). Therefore, the swimming defect in Δpili2 is likely due to impaired flagellar function or possibly an increased tendency for filament detachment.

Moreover, T4P are often associated with twitching motility (10, 14, 45). In *Liberibacter crescens*, for instance, the Tad pilus has been implicated in this type of surface-associated movement (46), though in other species, it is not necessarily required. To assess whether Tad pili mediate twitching in *B. diazoefficiens*, we examined this behavior in the pilus mutants. In agreement with the swimming assay results, only the Δpili2 mutant exhibited a defect in twitching motility, while Δpili1 and Δpili3 behaved like the WT (Fig. 4C and D).

Additionally, we observed that the Δpili2 mutant was unable to spread across the surface of agar plates (Fig. 4E and F), indicating the loss of SAM. The pattern observed was distinct from swarming, which is an active, flagellum-dependent, and coordinated multicellular movement over a surface. Instead, the phenotype observed here resembles the sliding type of motility, which is considered a passive mode of surface expansion that does not require flagella or active energy expenditure and is generally associated with surfactant production or growth-driven colony spreading (47). Nevertheless, we cannot exclude the possibility that Tad pili actively contribute to this colony expansion. Therefore, this behavior is termed SAM. The absence of SAM in the Δpili2 mutant provides further evidence of broader regulatory disruptions beyond structural defects, potentially involving alterations in cell envelope properties or signaling cascades. Importantly, this motility impairment was also observed in the double mutants Δpili1Δpili2 and Δpili3Δpili2 (see Fig. S8 at http://sedici.unlp.edu.ar/handle/10915/186424), reinforcing that the phenotype is specifically associated with the cluster 2 deletion.

Taken together, these results show that deletion of cluster 2 generates an impairment in all motility behaviors in *B. diazoefficiens* USDA 110, including swimming, twitching, and SAM. The comprehensive motility deficiency observed in the Δpili2 mutant suggests that TP2 contributes not only to pilus-dependent movement but also to broader regulatory networks, affecting flagellum-mediated swimming and SAM.

### Lack of Tad pili does not modify exopolysaccharides production

**Fig 5:**
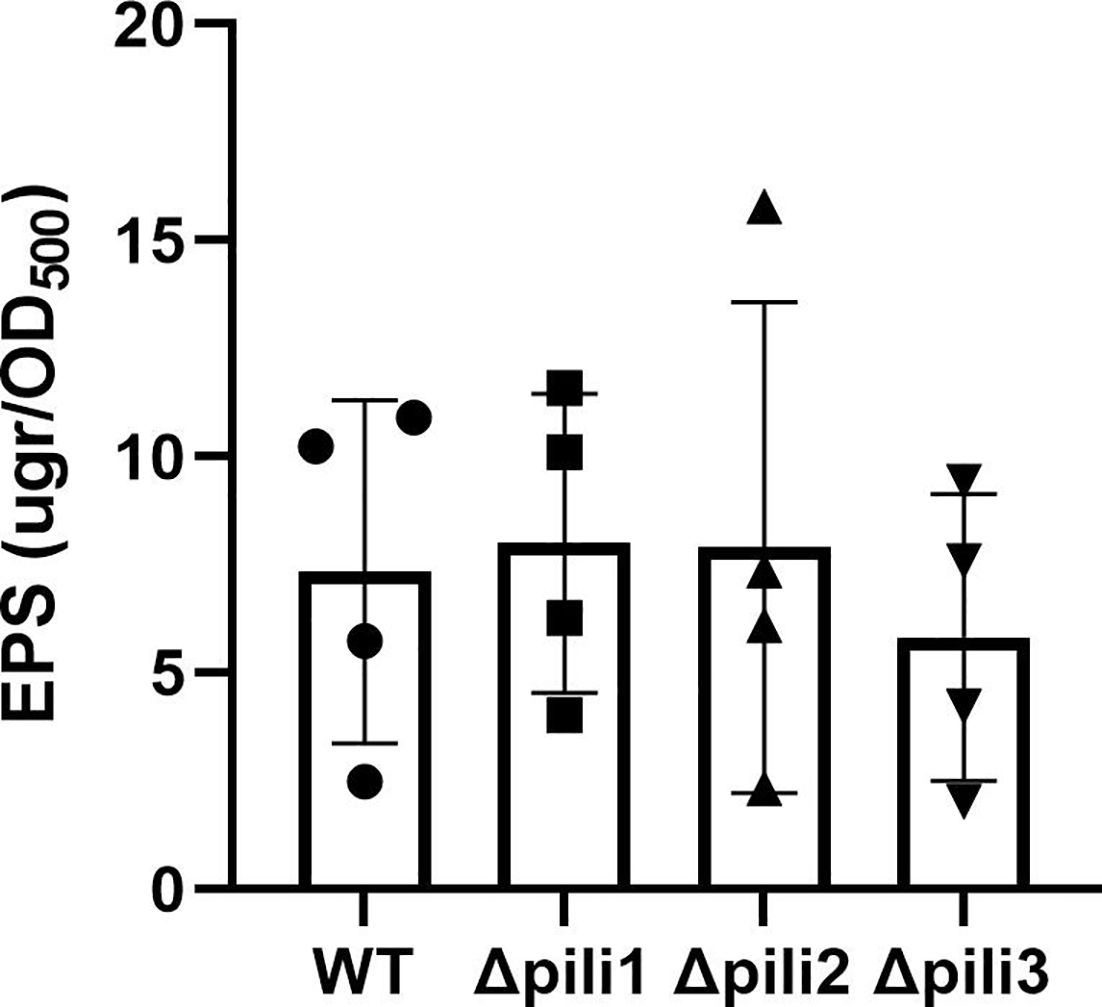
EPS quantification in WT and Δpili mutants. The amounts of EPS are expressed based on total culture growth (OD_500_). Data represent results from four independent experiments, each with three biological replicates. EPS values are given as means ± SD. No significant differences were found by ANOVA and Tukey’s *post hoc* test for comparisons between strains.

The quantity (or quality) of exopolysaccharides (EPS) produced may affect bacterial surface cell-cell interactions (48) and biofilm formation. To establish possible links between EPS production and the presence of each set of pili loci, we measured the amounts of EPS produced by all pili mutants compared to the WT strain. Figure 5 shows the results of four independent experiments where no significant differences between the mutant strains and the WT were observed. Therefore, the Δpili2 phenotype cannot be attributed to the alteration of EPS amounts produced by these strains.

### The Δpili2 mutant has augmented c-di-GMP level

**Fig 6:**
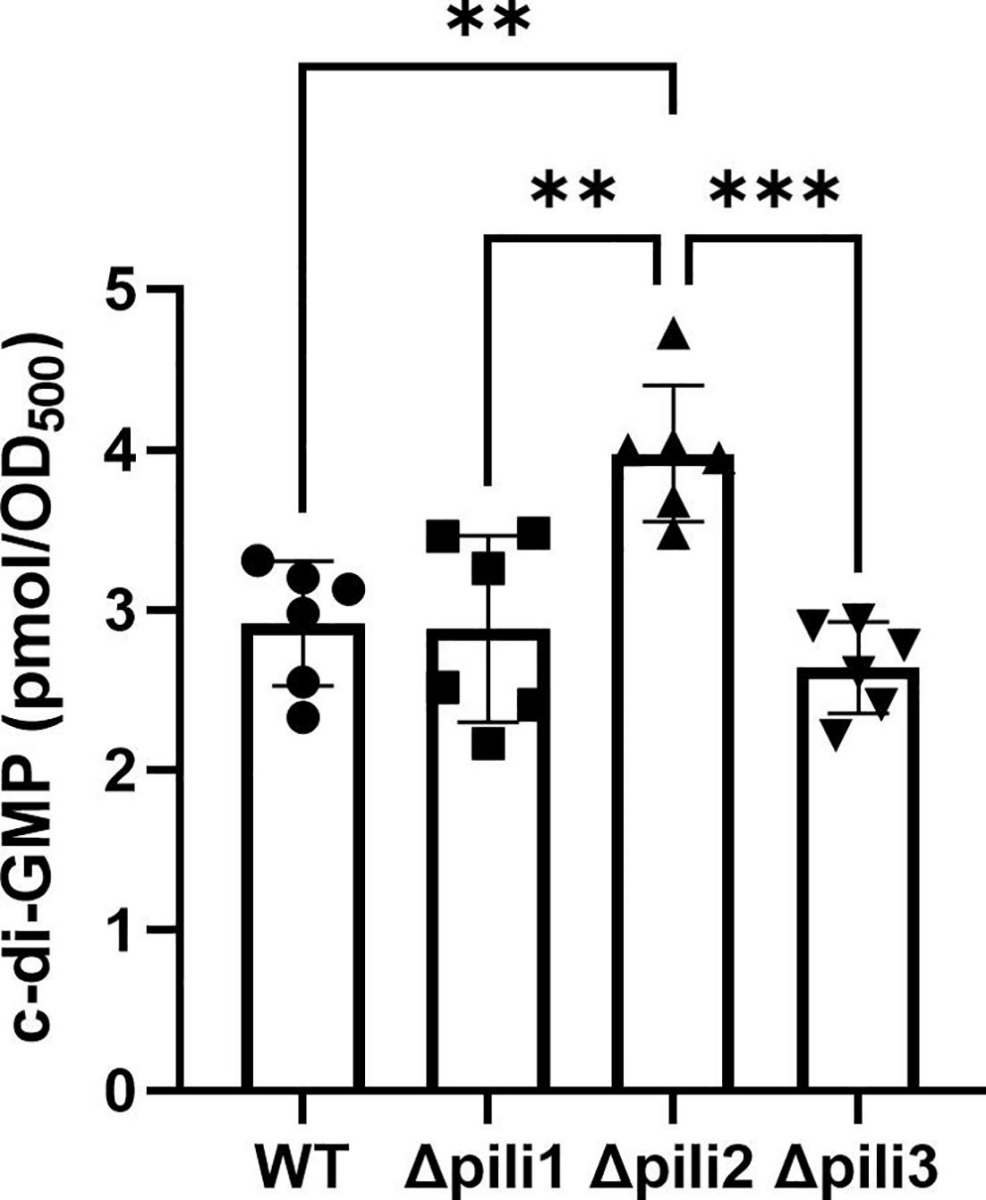
c-di-GMP quantification by HPLC-MS/MS identification. Data represent results from two independent experiments, each with three technical replicates. Statistical significance was determined by analysis of variance and Tukey’s *post hoc* test comparison for differences between strains indicated. **, *P* < 0.01 and ***, *P* < 0.001; only statistically significant differences have been indicated.

Up to this point, the phenotype observed in the Δpili2 mutant indicates an increase in its biofilm-forming capacity. In general, the biofilm state is associated with elevation of the intracellular concentration of the c-di-GMP second messenger pool (49–51). To test this relationship, we measured c-di-GMP levels in the WT and the Δpili mutants grown in liquid culture. As shown in Fig. 6, the intracellular level of c-di-GMP in the Δpili2 mutant was 36% higher than the WT, which suggests that the biofilm effects of this deletion might be mediated by second messenger levels.

### The Flp pilin subunit encoded in cluster 2 partially contributes to the Δpili2 mutant phenotype

**Fig 7:**
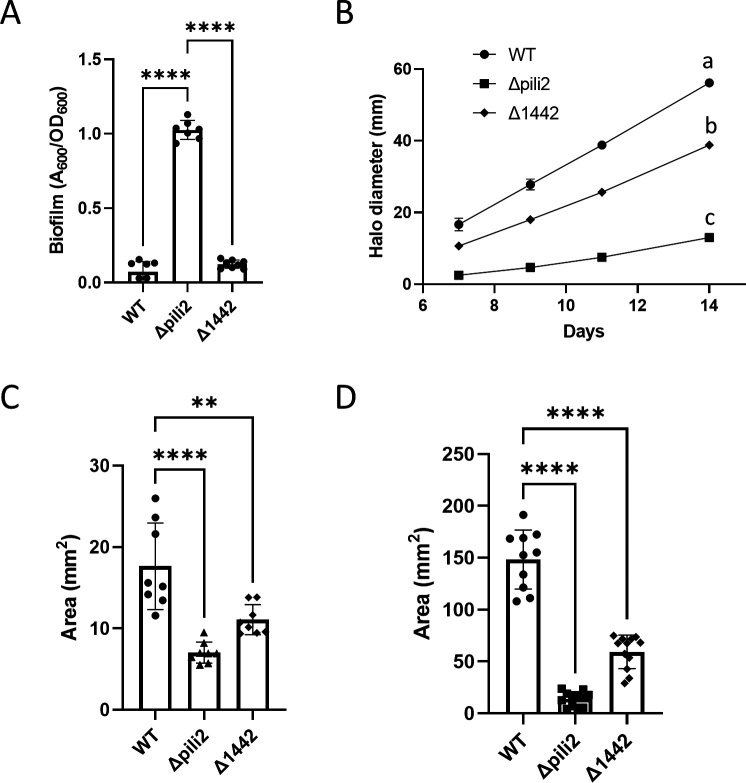
Biofilm formation and motility characterization by WT and Δpili2 and D1442 mutants. (**A**) Biofilm formation capacity of WT and Δpili mutants measured after 7 days of incubation. (**B**) Swimming in semisolid AG medium (0.3% agar) over time. (**C**) Area covered by twitching motility in the interface between the growth medium and the plate (AG medium, 0.8% agar). (**D**) Area covered by SAM over the surface of the growth medium (AG medium, 0.8% agar). Data represent results from at least two independent experiments, each with four biological replicates. Statistical significance was determined by analysis of variance and Tukey’s *post hoc* test comparison for differences between strains indicated. **, *P* < 0.01, **** or different letters indicate *P* < 0.0001; only statistically significant differences have been indicated.

In the Δpili2 mutant, most of the genes in cluster 2 were deleted; however, the gene encoding the Flp prepilin subunit (bsl1442) remained intact (see Fig. S5 at http://sedici.unlp.edu.ar/handle/10915/186424). This raised the possibility that bsl1442 might contribute to the phenotypes observed in the Δpili2 mutant. To assess its role, we generated a bsl1442 deletion mutant (Δ1442) in which the rest of cluster 2 remained unaltered (see Fig. S9 at http://sedici.unlp.edu.ar/handle/10915/186424). The Δ1442 strain exhibited a phenotype largely similar to WT, with biofilm formation levels comparable to WT and only a slight reduction in motility (Fig. 7).

This result suggests that Flp encoded by bsl1442 is not essential for biofilm formation under the tested conditions. It is plausible that other pilin-encoding paralogs (Fig. 1B) may compensate for the absence of bsl1442, maintaining functional assembly of the TP2 apparatus, as previously described in *A. tumefaciens* (26). However, the partial motility defect indicates that such compensation is not sufficient to fully restore WT function.

**Fig 8:**
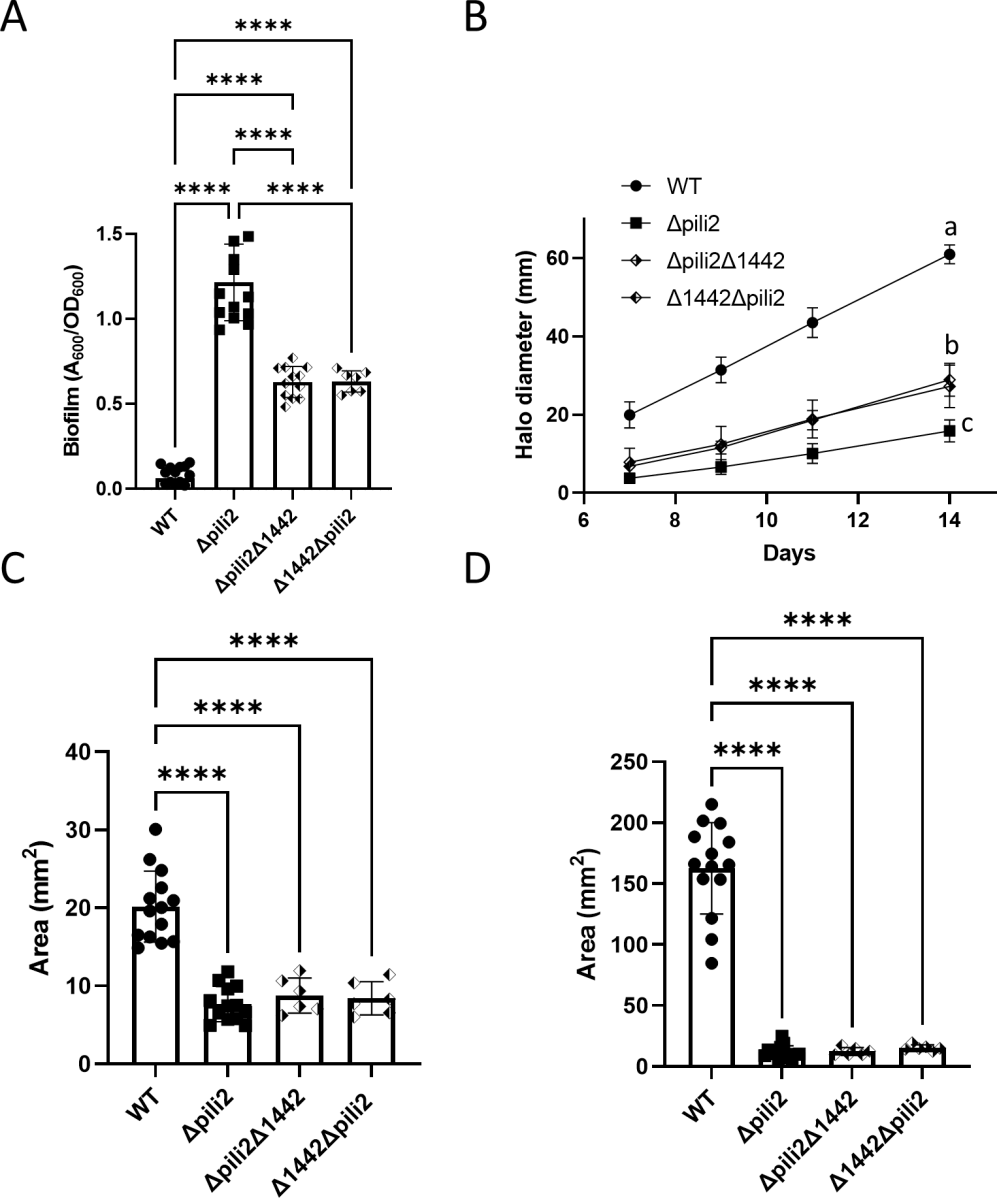
Biofilm formation and motility characterization by WT and Δpili2, Δ1442Δpili2, and Δpili2Δ1442 mutants. (**A**) Biofilm formation capacity of WT and Δpili mutants measured after 7 days of incubation. (**B**) Swimming in semisolid AG medium (0.3% agar) over time. (**C**) Area covered by twitching motility in the interface between the growth medium and the plate (AG medium, 0.8% agar). (**D**) Area covered by SAM over the surface of the growth medium (AG medium, 0.8% agar). Data represent results from at least two independent experiments, each with four biological replicates. Statistical significance was determined by analysis of variance and Tukey’s *post hoc* test comparison for differences between strains indicated. **** or different letters indicate *P* < 0.0001; only statistically significant differences have been indicated.

To further clarify the contribution of bsl1442 to the Δpili2 phenotype, we constructed two functionally equivalent double mutants: Δ1442Δpili2 and Δpili2Δ1442. Both strains lack the entire TP2 apparatus as well as the bsl1442 gene. Phenotypic analysis showed that these double mutants formed biofilms at levels intermediate between the WT and the Δpili2 mutant, while motility remained as impaired as in the Δpili2 strain (Fig. 8). These findings indicate that bsl1442 contributes partially to the Δpili2 phenotype but also point to additional bsl1442-independent mechanisms—potentially involving accumulation of unprocessed pilins or dysregulated signaling—that exacerbate the observed defects.

To assess whether the TP3 system could compensate in the absence of TP2 and bsl1442, we deleted bsl1442 in the Δpili3Δpili2 background, generating a triple mutant lacking both clusters 2 and 3. This strain exhibited a phenotype indistinguishable from the Δ1442Δpili2 and Δpili2Δ1442 strains (see Fig. S10 at http://sedici.unlp.edu.ar/handle/10915/186424), suggesting that the TP3 system does not contribute to the altered phenotype in the absence of TP2 and bsl1442. Together, these results support a role for bsl1442 in modulating motility and biofilm formation, while also implicating additional, yet unidentified regulatory or structural components in the control of these phenotypes.

### The lack of Tad pili does not affect soybean nodulation

One of the most remarkable features of *Bradyrhizobium* species is their ability to establish symbiotic interactions with legume hosts. During the early stages of root colonization by *B. diazoefficiens*, flagellar swimming motility has been shown to be dispensable under non-flooded conditions, and swarming motility appears to play no significant role in soil colonization (44, 52). In contrast, root adhesion is a critical prerequisite for infection and nodulation, and strains with enhanced adhesive capabilities exhibit increased competitiveness and infectivity (53, 54). Given the role of pili in surface attachment and cell-cell interactions, it is plausible that these structures could contribute to the symbiotic phase of the *B. diazoefficiens* life cycle.

**Fig 9:**
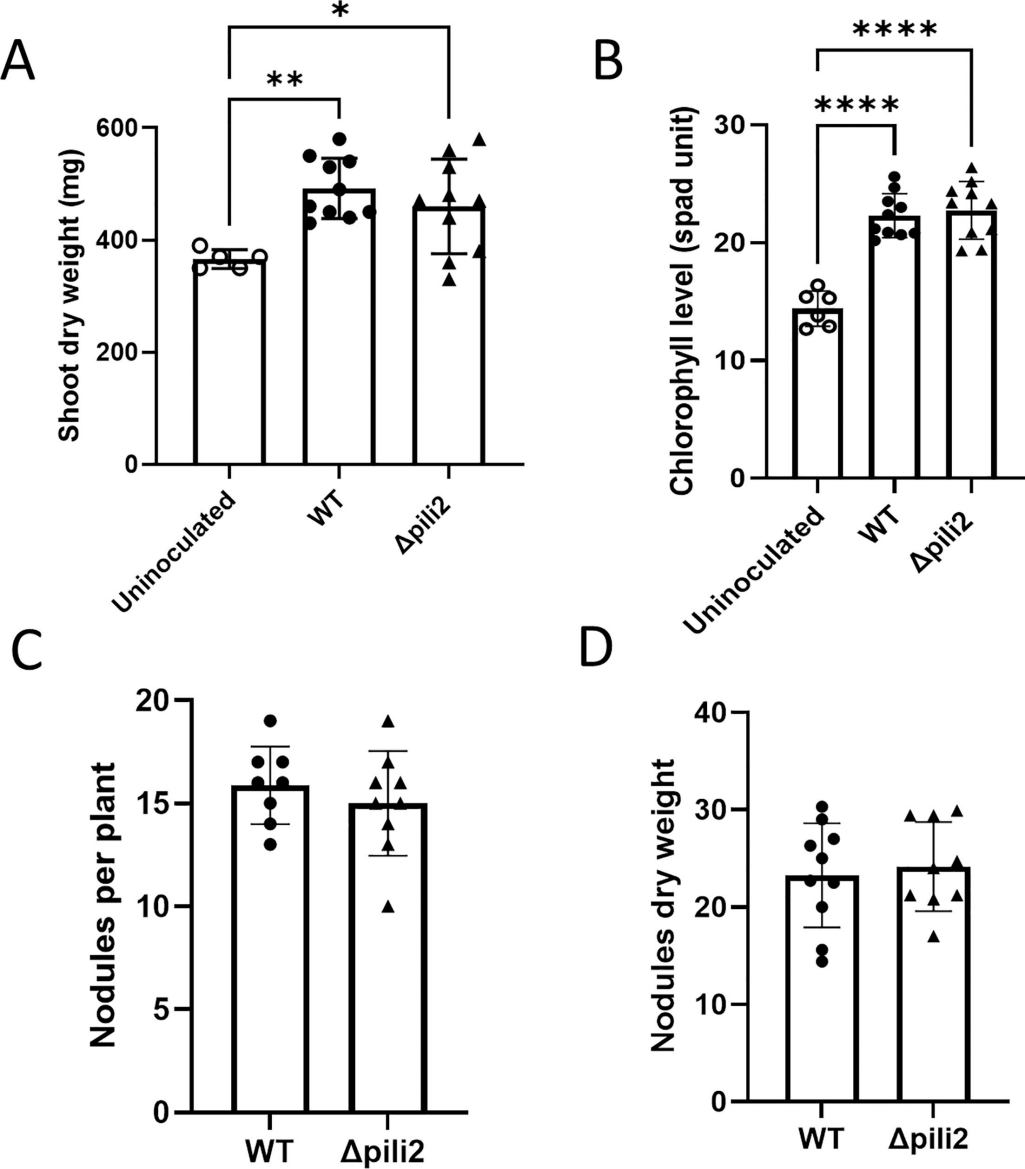
Pili mutant displays a normal symbiotic interaction with soybean. (**A**) Shoot dry weight per plant. (**B**) Chlorophyll content in leaves. (**C**) Number of nodules per plant. (**D**) Nodules dry weight per plant. Results correspond to one representative experiment from two independent repetitions, and all results are given as means ± SD. *, *P* < 0.05; **, *P* < 0.01; and ****, *P* < 0.0001; only statistically significant differences have been indicated.

To assess whether Tad pili are involved in the symbiotic process, we conducted nodulation assays using soybean plants inoculated with either the WT or the Δpili2 mutant. As shown in Fig. 9A through C, no significant differences were observed between the strains in any of the parameters measured, including shoot dry weight, leaf chlorophyll content, number of nodules, or nodule dry weight. These results demonstrate that the absence of the TP2 pilus does not impair the ability of *B. diazoefficiens* to successfully nodulate soybean plants under the conditions tested.

## DISCUSSION

This work focused on the characterization of the Tad pili apparatus in *B. diazoefficiens* USDA 110, which could be extended to other species in the *Bradyrhizobium* genus, thereby contributing to the understanding of its role in surface sensing, adhesion, and biofilm formation. Our findings highlight the significance of Tad pili in the lifestyle of *B. diazoefficiens*, shedding light on its multi-faceted role in bacterial physiology and potential applications in agriculture and biotechnology.

Our bioinformatic analysis revealed that Tad pili gene clusters are widely distributed and highly conserved across the *Bradyrhizobium* genus (see Fig. S2 at http://sedici.unlp.edu.ar/handle/10915/186424), suggesting a potentially important role in environmental adaptation—an aspect that has not yet been explored in this group. We identified multiple genomic clusters related to Tad pili, some of which appear to encode complete pilus systems, while others are partial or fragmented.

Clusters 1 and 2 showed the highest degree of conservation. Cluster 1 is present in all but one of the analyzed genomes, although it lacks certain key genes required to assemble a complete exporter apparatus, indicating that it may not function independently. In contrast, cluster 2 contains the full set of genes typically associated with a functional Tad pilus and is absent in only three genomes, highlighting its widespread distribution and potential functional relevance. Cluster 3 also encodes what appears to be a complete Tad pilus apparatus; however, it is present in only about half of the strains analyzed. Remarkably, only one strain carried cluster 3 in the absence of cluster 2, supporting the idea that cluster 2 plays a more fundamental or ancestral role in this genus. Phylogenetic analysis further supports this distinction, as clusters 2 and 3 form separate evolutionary lineages, suggesting they did not originate through a recent gene duplication event but rather through independent evolutionary trajectories. Cluster 4, which encodes minor pilins, is the least conserved in its canonical form and frequently appears fragmented. Nevertheless, homologs of its associated functions are found in nearly all genomes, at frequencies similar to those of clusters 1 and 2. This pattern points to a potentially essential role for these minor pilins in pilus assembly or function, consistent with previous findings in other bacteria that show they are required for full pilus activity (33). Moreover, our prior work demonstrated that mutations in cluster 4 genes impair root adhesion in *B. diazoefficiens*, further supporting their functional importance (29).

In other bacterial models, pilus loss typically impairs surface adhesion and biofilm formation (34–41, 43). In contrast, the Δpili2 mutant of *B. diazoefficiens* USDA 110, lacking most genes from cluster 2, exhibited a marked increase in biofilm formation. This unexpected phenotype suggests that disruption of the TP2 apparatus may trigger a compensatory regulatory response, potentially involving overexpression of alternative adhesins or loss of repression mechanisms, ultimately promoting a sessile, biofilm-forming lifestyle. This effect is not attributable to deregulation of either cluster 1 or cluster 3, since the double mutants Δpili1Δpili2 and Δpili3Δpili2 fail to suppress the enhanced biofilm phenotype. When we produced the Δpili2 mutant, we removed most of the genes encoded in cluster 2 but left intact *bsl1442*, annotated as Flp pilin, at the 3′ end of the cluster. This gene has its own promoter and was observed as expressed in liquid culture (55). In *C. crescentus*, one of the mechanisms that operates in the surface-sensing process involves the Tad pili and c-di-GMP signaling (21–23). In this way, the amount of mature pilin present in the plasma membrane appears to be one of the signals that activates the PleC histidine kinase pathway, generating an increase in the amount of intracellular c-di-GMP through the activation of the diguanylate cyclase, PleD (56). This is in agreement with the results obtained by Snyder et al. (57), who observed that the obstruction of the pili filament motion leads to an increase in the intracellular pool of c-di-GMP, along with premature replication initiation and an increase in stalk production. These observations lead to one of the accepted models in *C. crescentus* that postulates the function of Tad pili as a mechanical surface sensor (22). This model proposes that, when the retraction of the filament is blocked because it is attached to a surface, accumulation of mature pilin subunits in the plasma membrane takes place (21). This accumulation works as a signal that activates the synthesis of c-di-GMP and starts the cellular adhesion program (56). This increase in c-di-GMP has been linked to enhanced production of the holdfast in *C. crescentus* (22, 23) but also to the polar polysaccharide in *A. tumefaciens* (58), thereby triggering the transition toward biofilm formation. A similar mechanism has been described in *P. aeruginosa*, where elevated c-di-GMP levels are induced by surface sensing mediated by Type IVa pili or the flagellar system, leading to the activation of adhesion mechanisms (59, 60).

In the Δpili2 mutant, the secretion apparatus is inoperative, preventing the assembly and extrusion of Flp-type pilins. As a result, Bsl1442 is likely to accumulate in the plasma membrane, acting as a strong signal that stimulates c-di-GMP synthesis and leads to enhanced biofilm formation, as observed in microtiter plate assays. This increase in the second messenger may mediate the stimulation of polar polysaccharide production (61) or other types of adhesins, similar to what occurs in *C. crescentus* and *A. tumefaciens*.

To evaluate the role of Bsl1442 in this process, we generated the double mutant Δpili2Δ1442, which lacks both the secretion system and the major pilin. This strain exhibited a significant reduction in biofilm formation compared to Δpili2, though not to WT levels. The intermediate phenotype suggests that Bsl1442 is the main contributor to the c-di-GMP signal in the Δpili2 background, but other Flp-like proteins may also play a role. Indeed, the *B. diazoefficiens* genome encodes five additional *flp* homologs outside the canonical clusters, which may accumulate in the absence of TP2 and weakly activate c-di-GMP signaling. However, their individual contribution appears much lower than that of Bsl1442, consistent with the partial phenotype of the double mutant. Other pili-independent pathways may also occur. In *Caulobacter*, a diguanylate cyclase associated with the flagellar motor has been implicated in the c-di-GMP signaling during the transition to surface attachment (24). *B. diazoefficiens* expresses two independent flagellar systems, yet the potential surface-sensing mechanisms operating in each apparatus remain unknown.

Importantly, the Δ1442 single mutant did not show increased biofilm formation, indicating that in the presence of a functional secretion system, Flp proteins are exported efficiently and do not trigger c-di-GMP signaling. Moreover, deletion of both clusters 2 and 3 (Δpili3Δpili2Δ1442) did not further reduce biofilm formation compared to the Δpili2Δ1442 double mutant. This rules out a role for cluster 3 in the enhanced biofilm phenotype of Δpili2, reinforcing the conclusion that the phenotype arises from the loss of TP2 function and the accumulation of Flp-type pilins—particularly Bsl1442—rather than from the activity of cluster 3.

A striking effect of the Δpili2 mutation was the inhibition of bacterial autoaggregation, as cells remained in suspension longer compared with the WT and the Δpili1 and Δpili3 mutants (see Fig. S6 at http://sedici.unlp.edu.ar/handle/10915/186424). In PVP multiwell cultures, the WT strain clearly tended to autoaggregate and sediment (resulting in a clear bottom at the base of the well) rather than adhere to the wall (with almost no ring visible in the CV assay). In *Bordetella holmesii*, a mutant in BipA, a protein that inhibits autoaggregation, produces an augmented speed of sedimentation and therefore an inhibition in biofilm formation, which agrees with our hypothesis (62). In contrast, the absence of sedimentation in the Δpili2 mutant may contribute to its enhanced biofilm formation, evidenced by a pronounced CV ring near the top of the well. The loss of functional TP2 pili (Δpili2 mutant), together with the Flp signal (pilin accumulation in the membrane), may alter cell surface properties and expose alternative adhesion molecules (likely the unipolar polysaccharide), thereby promoting increased adhesion to the well surface. These observations suggest that initial adhesion to a surface, which ultimately leads to the development of a mature biofilm, is regulated by multiple signals and requires a fine balance among them to achieve the final outcome. Taken together, all these results and those informed in the present paper point to a complex interaction among pili functions in *B. diazoefficiens*, where at least cluster 2 might mediate cell-cell contacts required for cell-to-cell autoaggregation. This hypothesis agrees with the CLSM images in Fig. 3, where it is evident that the WT formed more compact structures than the Δpili2 mutant, and in mixed inocula, both strains were mutually excluded, perhaps because of the inability of the Δpili2 mutant to recognize some target in the cell surface for autoaggregation. This intricate interaction among pili clusters might be related to the complexity of the free-living state of this bacterium, characterized by the need to settle on surfaces such as soil particles and organic remnants for survival in the soil, or on root surfaces to initiate infections.

While the pilus apparatus should not be directly involved in swimming or SAM, it could exert some kind of direct or indirect control in *B. diazoefficiens* USDA 110. The impaired motility of the Δpili2 mutant is in agreement with its augmented biofilm formation capacity. Both characteristics could be explained by the increased level in the intracellular c-di-GMP pool in the mutant.

Notably, soybean nodulation and N₂ fixation were not affected by TP2 deletion (Fig. 9). This suggests a more significant function of this system in free-living bacteria; however, more specific parameters such as competitiveness for nodulation or infectivity will need to be revised in the future.

In conclusion, our study provides valuable insights into the genetic and functional landscape of Tad pili in *B. diazoefficiens*. The diverse roles and complex interactions of these pili in cell-to-cell autoaggregation and biofilm development highlight their importance in bacterial survival and adaptation to symbiosis. Understanding these mechanisms opens avenues for potential applications in enhancing bacterial symbiosis with plants, improving agricultural productivity, and developing novel strategies for biofilm management in industrial settings. Future research should focus on elucidating the specific regulatory pathways and environmental signals that modulate Tad pili functions, further enhancing our understanding of bacterial physiology and its applications.

## MATERIALS AND METHODS

### Bacterial strains and culture conditions

The bacterial strains and plasmids used in this work are summarized in Table S4 (http://sedici.unlp.edu.ar/handle/10915/186424). All *B. diazoefficiens* strains were routinely grown on solid yeast extract-mannitol medium (63) at 28°C. For all assays, the strains were grown in arabinose-gluconate-supplemented HM salts (AG medium) at 28°C and 180 rpm (64). For conjugation assays, bradyrhizobia were grown in peptone-salt-yeast extract medium (65). *Escherichia coli* strains were grown in Luria-Bertani medium (66) at 37°C. Antibiotics were added at the following concentrations: 150 and 25 µg/mL kanamycin (Km), 100 and 10 µg/mL gentamicin (Gm), and 100 and 10 µg/mL tetracycline (Tc) for *B. diazoefficiens* and *E. coli*, respectively; 200 µg/mL ampicillin (Ap) for *E. coli*; and 20 µg/mL chloramphenicol (Cm) for *B. diazoefficiens*.

### Cloning procedures

All cloning procedures, including DNA isolation, digestion, ligation, and strain transformation, were carried out according to Sambrook and Russell (66). To generate the Tad pili deletions in *B. diazoefficiens* USDA 110, a markerless strategy based on the pK18mobSacB plasmid was used (67). The vectors needed for mutant construction were obtained by cloning upstream and downstream DNA fragments of the region targeted for deletion, as indicated in Fig. S5 (http://sedici.unlp.edu.ar/handle/10915/186424). The exact position of the PCR fragments and the restriction enzymes used for this purpose is detailed in Fig. S5 (http://sedici.unlp.edu.ar/handle/10915/186424). PCR reactions were carried out using Taq or Pfu polymerase (Productos Bio-Lógicos, Quilmes, Argentina), and the primers used are listed in Table S5 (http://sedici.unlp.edu.ar/handle/10915/186424). PCR fragments were purified from agarose gels using an extraction kit (DSBIOTM) and digested with the appropriate restriction enzymes (Promega) according to the manufacturer’s instructions. The pK18mobsacB::Δpili1, pK18mobsacB::Δpili2, and pK18mobsacB::Δpili3 plasmids (see Table S5 at http://sedici.unlp.edu.ar/handle/10915/186424) were transferred by conjugation from *E. coli* S17-1 to *B. diazoefficiens* USDA 110 as previously described (67). Merodiploids for each recombination event (upstream and downstream homologous fragments) were selected by kanamycin resistance and confirmed by PCR. To obtain deletion mutants, excision of the integrated plasmid from Km^R^, Suc^S^ clones was facilitated by culturing the recombinants in AG medium without antibiotics for 24–48 h, followed by plating on YME agar supplemented with 12.5% (wt/vol) sucrose. The resulting clones (kanamycin-sensitive and sucrose-resistant) were PCR-verified for the deletion event. The construction of strains with multiple deletions followed the same procedure but was applied to the desired genetic backgrounds. To obtain strains labeled with bjGFP or mCherry, the plasmids designed by Ledermann et al. (68) were used.

### Biofilm assay (adhesion to PVC 96-well plates)

Static biofilm formation was determined macroscopically by a quantitative assay in 96-well microtiter plates, with CV staining based on a method described previously by O’Toole and Kolter (45), with minor modifications. Bacteria were grown in 10 mL AG medium with agitation for 48 h at 28°C. Cultures were then diluted in fresh medium to an OD_₅₀₀_ of 0.05. A 150 µL aliquot of the suspension was added to each well and incubated at 28°C in a humidity chamber to prevent evaporation. Bacterial growth was quantified by measuring OD_₆₀₀_. Planktonic cells were gently removed, and each well was washed twice with 200 µL of distilled water and dried for 15 min in an incubator at 65°C. Then, 200 µL of 0.1% (wt/vol) aqueous CV solution was added and incubated for 15 min. CV-stained wells were rinsed thoroughly with water and then scored for biofilm formation by adding 200 µL of 33% acetic acid. The OD_₆₀₀_ of solubilized CV (150 µL) was measured using a microplate reader (Benchmark Plus by Bio-Rad with Microplate Manager 5.2.1 software or FLUOstar OPTIMA by BMG Labtech with OPTIME 2.20R2 software). Sterile control cultures with AG medium were included in each experiment. The estimated amount of adhered biomass was calculated by subtracting blank values and normalizing to the OD_₆₀₀_ of the planktonic culture.

### Microscopy analysis

Cells adhered to glass slides were treated as in the biofilm assay. Images were taken at 600× magnification using a Nikon Eclipse E400 microscope equipped with a Nikon Coolpix 4500 digital camera.

### CLSM imaging of biofilms

Biofilms were formed on chambered cover glass (Nunc Lab-Tek) at 28°C in a 7-day experiment to observe the architecture of single-strain and dual-strain biofilms. GFP-labeled and mCherry-labeled derivatives of WT and the Δpili2 mutant strains were used. Inocula were grown in 3 mL AG medium with agitation for 48 h at 28°C and then diluted to a final OD_₅₀₀_ of 0.1. A 250 µL volume of the suspension was added to microchamber wells and supplemented with 250 µL of fresh sterile AG medium to reach a final volume of 500 µL. Dual-strain cultures were inoculated with 250 µL of each strain at a 1:1 ratio. Microchambers were incubated statically at 28°C in a sterile humid container for 7 days. Confocal images were acquired using a Leica TCS SP5 microscope with a 40×/1.5 oil-immersion objective. Excitation wavelengths were 488 nm for GFP and 594 nm for mCherry, and fluorescence emission was collected at 490–550 nm and 580–650 nm, respectively. Optical sections were taken at 0.25 µm intervals to create z-stacks covering the full biofilm. Image processing was done using the open-source ImageJ/Fiji 6.3 software (69).

### Motility characterization

Swimming motility assays were performed on semisolid AG plates (25 mL of 0.3% [wt/vol] agar in 90 mm plates), inoculated with sterile toothpicks from solid cultures. Plates were incubated at 28°C, and the diameter of the motility halo was recorded for 2 weeks. For twitching and SAM assays, semisolid AG plates (0.8% agar) were inoculated by stabbing through the surface until reaching the bottom. SAM was recorded first. Then, the colony was carefully removed with water, and the bottom of the plate was photographed. ImageJ was used to measure the area occupied by each type of movement.

### Flagellin purification and analysis

For flagellin preparation, *B. diazoefficiens* was grown in 30 mL of AG liquid medium until OD_₅₀₀_ = 2. Supernatants were precipitated as described elsewhere (44), and samples were resuspended in the same volume. Samples were analyzed by SDS-PAGE as previously described (70).

### EPS quantification

AG cultures (30 mL) were harvested after 48 h of incubation at 28°C with agitation at 180 rpm (final OD_₅₀₀_ of 2). EPS was purified and quantified as previously described (48). For quantification, a colorimetric method using 0.2% (wt/vol) anthrone reagent in 96% sulfuric acid was used, with glucose (1 mg/mL) as the standard.

### Endometabolome extraction and c-di-GMP quantification

Each strain was grown for 48 h in AG medium at 28°C and 180 rpm. For endometabolome extraction, 5 mL of each culture was centrifuged at −10°C for 10 min at 5,000 rpm, with two biological and three technical replicates per strain. Pellets were frozen at −80°C until extraction. Cell lysis was performed using an ice-cold solution containing equal volumes of methanol (LC-MS grade) and TE buffer (10 mM Tris, 1 mM EDTA, pH 7) at a ratio of 500 µL per 5 mL of culture (OD_₅₀₀_ = 2). An equal volume of ice-cold chloroform was added, vortexed thoroughly, and incubated for 2 h at 0°C in a ThermoMixer at 1,000 rpm. Samples were centrifuged at −10°C for 10 min at maximum speed. The aqueous phase was filtered through 13 mm PTFE 0.2 µm hydrophobic syringe filters (Fisherbrand) and stored at −80°C until analysis.

### Quantitative determination of c-di-GMP was performed using LC-MS/MS

Chromatographic separation was done on an Agilent Infinity II 1290 HPLC system with a SeQuant ZIC-pHILIC column (150 × 2.1 mm, 5 µm, Merck) and guard column (20 × 2.1 mm, 5 µm, Phenomenex), at 40°C and 0.1 mL/min. Mobile phases were as follows: A, 10 mM ammonium acetate in water (pH 9) with 5 µM medronic acid and B, 10 mM ammonium acetate in 90:10 acetonitrile:water (pH 9) with 5 µM medronic acid. Injection volume: 5 µL. The mobile phase profile included 0–1 min 75% B; 1–6 min, 75%–40% B; 6–9 min, 40% B; 9–9.1 min, 40%–75% B; 9.1–20 min, 75% B. Detection was done with an Agilent 6495 ion funnel mass spectrometer using ESI (positive and negative modes) under standard conditions. Compound identification was based on mass transition and retention time compared to a chemical standard. Chromatograms were integrated using MassHunter (Agilent). Absolute concentrations were determined from an external standard curve.

### Bioinformatics methods and analysis

Tad pili clusters in *B. diazoefficiens* were identified using NCBI BLASTn (71), with reference tad clusters from *S. meliloti* 2011, *A. tumefaciens* C58, *C. crescentus* N1000, and *A. actinomycetemcomitans* D7S-1. Functional assignment was based on bidirectional BLASTp with the same references. Cluster conservation was analyzed with Clinker (72). A custom pipeline was used to search for tad clusters in other *Bradyrhizobium* species using Python scripts. Genomes were downloaded in GenBank format with download_gb_from_tsv.py, converted to FASTA using gb_files_to_fasta.py, and queried with blast_contigous_segments.py, which merges nearby HSPs. Scripts are available at GitHub. Figure S2 (http://sedici.unlp.edu.ar/handle/10915/186424) was generated using the clustermap function from the Seaborn Python library.

### Plant assay

DonMario 4800 soybean seeds were surface-sterilized by immersion in 96% (vol/vol) ethanol for 5 s, then in 20% (vol/vol) commercial bleach for 10 min, followed by six washes in sterile distilled water. Seeds were germinated on 1.5% (wt/vol) agar in the dark at 28°C. Nodulation assays were conducted in vermiculite-filled pots initially irrigated with Fåhraeus mineral solution. After 30 days at 26°C under a 16 h photoperiod, the number of nodules and the dry weight of shoots and nodules per plant were determined. Leaf chlorophyll content was measured using a SPAD-502 chlorophyll meter (Minolta, Japan) on each leaflet of a trifoliate leaf. Combined SPAD readings were averaged for each trifoliate leaf (73).

### Statistical analysis

Each experiment included at least two biological replicates (as indicated in each experiment). Data were analyzed for statistical significance using a one-way ANOVA followed by Tukey’s multiple-comparison test to assess differences among groups. The significance level is specified in the figure legend of each experiment.

## Data Availability

Supporting information is available at http://sedici.unlp.edu.ar/handle/10915/186424.
